# Association of Musculoskeletal and Radiological Features with Clinical and Serological Findings in Systemic Sclerosis: A Single-Centre Registry Study

**DOI:** 10.31138/mjr.31.3.341

**Published:** 2020-09-30

**Authors:** Nasrin Azarbani, Ali Javadzadeh, Iman Mohseni, Arash Jalali, Elham Andalib, Hadi Poormoghim

**Affiliations:** 1 Arak University of Medical Sciences, Arak, Iran; 2 Iran University of Medical Sciences, Tehran, Iran; 3 Tehran University of Medical Sciences, Tehran, Iran

**Keywords:** systemic sclerosis, disease subsets, musculoskeletal symptoms, hand radiography, joint erosion

## Abstract

**Aim:**

Systemic sclerosis (SSc) is a chronic connective tissue disease with the clinical hallmark of skin thickening and tethering. Correlation of musculoskeletal features with other parameters should be considered in SSc patients.

**Methods:**

We reviewed the records of all patients who had more than one visit and standard anteroposterior radiography of hand. We used univariate analysis, and factors with p<0.05 were included in logistic regression to find out dependent factors.

**Results:**

Overall, 180 SSc patients were enrolled in our study, 161 (89.4%) of whom were women. Median age (IQR) was 47.0 years (16), and 52% had diffuse subtype of the disease. In multivariate analysis, tendon friction rubs (TFRs) was associated with the presence of calcinosis, muscle tenderness, and flexion contracture (FC) on physical examination (p<0.05). Arthritis showed no differences in the two subtypes of the disease (p=0.98), and in multivariate analysis, there were no correlations between radiographic arthritis and serological and clinical features. The radiographic results indicated that disease duration correlated with joint erosion, acro-osteolysis, resorption of distal ulna, calcinosis and radiologic FC (p< 0.05). Acro-osteolysis was more frequent in the dcSSc subtype, TFRs, and anti-TOPO I antibody. Radiologic FC showed association with skin score, calcinosis and haematocrit <30% (p<0.05). Joint flexion on radiography was associated with disease duration, modified Rodnan skin score, calcinosis, and low haematocrit (P<0.01).

**Conclusion:**

Disease duration was a main dependent factor for developing joint erosion, acro-osteolysis, bone resorption, calcinosis, and flexion contracture on hand radiography. Acro-osteolysis presented in the severe form of the disease. Acro-osteolysis was the only dependent variable associated with bone demineralization.

## INTRODUCTION

Systemic sclerosis (SSc) is a devastating connective tissue disease characterized by fibrosis, autoimmunity, and vascular changes that affect skin and visceral organs. Musculoskeletal (MSK) manifestations of SSc vary from arthralgia that is reported in 23–81% of the cases^1–4^ to arthritis on physical examination reported in 5–68% of the cases.^5–12^ Muscles are affected in SSc, and patients could present with muscle pain, atrophy, and weakness. Other musculoskeletal manifestations of SSc include calcinosis and tendon friction rubs (TFRs) or rupture due to tendon sheath inflammation or fibrosis.^13^ Hand arthritis in conjunction with Raynaud’s phenomenon could be an early symptom of SSc.^14^ Physicians should be aware of hand symptoms, such as synovitis, joint erosions on fingers, skin thickening with tethering leading to flexion contracture and limitation of hands’ motion, as early symptoms of SSc. These symptoms may affect daily life activities and dexterity.^15,16^ Previous studies on SSc hand involvement have shown remarkable changes on radiography.^5–7^ Radiological patterns of joint abnormality are defined as erosion, narrowing, and arthritis, and bone and soft tissue patterns include demineralization, bone resorption, acro-osteolysis, calcinosis, and flexion contracture.^16^

Although SSc is a rare disease, MSK involvement is a common manifestation with a variable degree of prevalence. European Scleroderma Trials and Research group (EUSTAR) studies, which focus on clinical, radiological and serological features of MSK manifestation in SSc, also reported the association of these features with other measurement/phenotypes of the disease.^5,6,17–19^ There is only one study with regards to the correlation of radiological and MSK manifestations of SSc with other organs.^5^ In the current study, our goal was to first determine the point prevalence of the MSK manifestation in SSc patients, and secondly, to evaluate MSK manifestation’s correlation with other disease parameters.

## METHODS

In a scleroderma cohort study, we reviewed the medical records of all registered SSc patients in Firoozgar Hospital’s database, who had more than one visit and had standard anteroposterior radiography of their wrist and hand. All the basic and para-clinical data was extracted from scleroderma registry in our clinic. The patients were included if they met the American College of Rheumatology/European League Against Rheumatism (ACR/EULAR 2013) classification criteria for SSc.^21^ Disease subset distinction was conducted according to Le Roy criteria.^22^

Patients’ data extracted from the database included: age, gender, skin score (based on modified Rodnan’s Skin Scoring, mRSS), disease duration (the time of first non-Raynaud symptom to the time of the radiographic study), the presence of active or healed digital ulcer, telangiectasia, amputation, and calcinosis. Musculoskeletal involvement is defined in **Table 1**.

**Table 1.** Definition of musculoskeletal involvement in systemic sclerosis in 180 patients with systemic sclerosis.

**Joint and tendons involvemen**t
Synovitis: swelling and tenderness on >1 joint
Tendons friction rub: palpable leathery sensation on active or passive motion of tendons
Joint contracture defined as uncorrected deformity of the joint usually in flexion position with decreased range of motion and prevented full extension of joint
*Flexion contracture on hand: the presence of prayers sign in physical examination and Finger to Palm Distance (FTP). It measured by the distance between the tip of the 3rd finger on dominant hand to distal palmar crease on full flexion by patients. ^23^ The severity of flexion contracture is classified according to Medsger severity scale. ^24^

**Muscular Involvement**

Muscular involvement was recorded as muscle atrophy, muscle tenderness, and muscle weakness, according to the Oxford scale (Muscle strength is graded 0 to 5)

*Preliminary SSc severity scale from reference (finger to palm distance, FTP on full flexion): FTP 0–0.09 cm (normal); FTP 1.0–1.9 cm (mild); FTP 2.0–3.9 cm (moderate); FTP 4.0–4.9 cm (severe); FTP 5.0+ cm(end stage) ^24^

### Radiographic study

As the first step, two specialists including one radiologist (I.M.) and one rheumatologist (A.J.) independently carried out a radiological assessment of the hands and wrists according to a predefined pattern. They were blinded to the patients’ clinical data. Afterwards, if the assessments were not consistent between the two evaluators, then a second review was requested in order to reach a consensus with regards to the clinical decision for further evaluation.

### Serologic studies

Erythrocytes sedimentation rate (ESR) more than 30 mm/1 hr and CRP> 10 mg/ml were considered as abnormal results. Muscle enzymes abnormality defined as CPK > 2 times of the normal level and aldolase more than 10 unit/ml. Also, ANA titer > 1:100 was considered as positive, and ANA patterns in sera were detected by the indirect immunofluorescence technique via Mosaic HEp-20-10 Liver (monkey). Anti-centromere Abs (ACA) and anti-Topoisomerase I Abs (anti-TOPO I abs) were detected by line immunoassay (Euroline systemic sclerosis profile [IgG], Euroimmune, Lubeck, Germany).

### Statistical analysis

Chi-square and Student’s t-test were performed for comparison of categorical and continuous data in the two subsets of disease. In the absence of normality assumption, Kruskal-Wallis test and pairwise comparison were used for comparing statistically significant differences in more than two independent groups. To find out the association between the two nominal variables, we used Cramer’s V coefficient. We used univariate analysis for the evaluation of the association between each MSK feature and demographic variables, other organ features, disease duration, and serologic data. Factors that showed significant association (p<0.05) in univariate analysis were used in linear logistic regression to assess dependent factors for each MSK feature.

### Ethics approval

The study was approved by the Human Research Ethics Committee of the research institution at Iran University of Medical Sciences (IUMS) in accordance with the ethical standards set forth in the 1964 Declaration of Helsinki and its subsequent amendments. Written informed consent was obtained from all the participants.

## RESULTS

### Study population

A total of 180 SSc patients were enrolled in our study, 161 (89.4%) of whom were women. Median age (IQR) was 47.0 (16) years. Ninety-five (52%) patients had a diffuse subtype of the disease. Mean modified Rodnan’s skin score of the patients was 10.54±8.80; mean disease duration was 6.00 ±5.02 years (**Table 2**).

**Table 2.** Demographic, clinical, and serological findings in 180 SSc patients.

Characteristic^*^	N (%)^**^
Gender: female	161(89.4)
Age >50	51(28.3)
Diffuse	95(52.8)
Rodnan skin score mean±SD	10.54±8.80
Duration from non-Raynaud’s symptoms to time of taken radiography mean years±SD	6.00 5.02
Raynaud’s phenomenon	164(91.1)
telangiectasia/face, hand, lip	129(71.1)
Hand telangiectasia	100(55.6)
Digital ulcer /gangrene	56(31.1)
Calcinosis of wrist and hand on PE	34(18.9)
Flexion contracture on physical examination (^¶^FTP > 0.9 cm)	101(55.2)
Arthritis on physical examination	35(19.4)
Tendon friction rub	41(22.8)
Muscle atrophy	60(33.3)
Muscle tenderness	37(20.6)
Muscle weakness	51(28.3)
ESR>30 (N=175)	49 (27.8)
CRP>10 (N=176)	31 (19.1)
RF + (N=165)	31 (18.8)
Anti-CCP + (No=141)	15(11.3)
ANA + (N=165)	153(92.7)
Anti-TOPO I (N=154)	100(64.9)
ACA + (N=154)	16(10.4)

* For Characters with missing value Number (No) of available data for analysis were recorded, other characters without missing value the number were not recorded.

** Number (percentage) for nominal data. Mean standard deviation (SD) for continuous data.

¶ FTP: finger to palm distance

## CLINICAL MANIFESTATIONS OF THE MUSCULOSKELETAL SYSTEM AND THEIR ASSOCIATION WITH OTHER FEATURES OF SSC

### Articular involvement

Arthritis or arthralgia, as the first presenting symptom, was recorded in 6.7% of the patients. On physical examination, arthritis > 1 joint was found in 35 (19.4%) of the patients, without significant difference with regards to the frequency of the symptom between the diffuse cutaneous systemic sclerosis (dcSSc) and limited cutaneous (lcSSc) subgroups.

### Joint contractures

Prayer sign on physical exam was present in 96 (55.2%) of the SSc patients and it was more frequent in patients with dcSSc subtype compared to lcSSc (67:96 [72%] vs. 20:85 [35.5], p<0.001). Mean FTP distance measurement was 2.131.58 cm in dcSSc and 1.141.24 cm in the lcSSc subtype (p<0.001).

Kruskal-Wallis test showed a significant difference in mRSS across categories of severity scales of FTP (H = 44.51, p <0.001). In pairwise comparisons, we found significant differences among patients with normal contracture compared to patients with mild (p=0.015), moderate (p<0.001), severe (0.001) and end-stage (<0.001) disease based on the FTP severity scale and skin score.

### Tendon friction rubs

Tendon friction rubs (TFR)s were found in 41 (22.7%) of the patients, (27[65.8%] with dcSSc, 14 [34.1%] with lcSSc subtype). The results showed a trend towards higher prevalence in dcSSc patients, but the difference was not significant (p=0.07).

### Muscle involvement

Muscle tenderness on palpitation was recorded in 37 (20.6%) patients (25/37 [67.5 %] with dcSSc, 12/37 [32.4%] with lcSSc; p=0.04). Muscle atrophy was detected in 60 (33.3 %) patients and muscle weakness in 51 (28.3%). Muscle atrophy and muscle weakness showed no significant differences in the two groups of patients.

### Muscle enzyme

CPK was checked in 169 (94%) patients and elevated CPK was detected in 21(12.4%) patients. Elevated CPK showed significant differences with muscle weakness status (66.7% with muscle weakness vs 33.3% without muscle weakness, [p=0.001]). Results of aldolase were available in 103 (57.2%) patients and its elevated level was detected in 8 (7.8%) patients. High aldolase was detected in 62.5% of the patients with muscle weakness versus 37.5% without muscle weakness (p=0.07). Coincident elevation of aldolase and CPK was detected in 63.2% of the patients with muscle weakness on physical examination. Elevated CPK and aldolase showed no significant difference in the two subtypes of the disease. As shown in **Table 3,** correlation of TFRs was shown in univariate and multivariate analysis. TFRs in SSc patients was dependent on the presence of calcinosis on physical examination (p=0.01), muscle tenderness (p=0.02), and flexion contracture on physical examination (0.003).

**Table 3.** Association of Tendon Friction Rubs symptoms with clinical, radiological and laboratory parameters in 180 SSc patients.

	**Univariate analysis**	OR (95%CI)*		**Multivariate analysis**	**B**	**OR (95%CI)**	**p**
Tendon Friction Rubs	** Duration of disease from non-Raynaud flexion contracture of joint on ^#^ PE calcinosis on ^#^ PE muscle pain/ tenderness muscle weakness muscle atrophy arthritis on PE low hematocrit high ESR acro-osteolysis on radiography bone resorption on radiography, FC on radiography arthritis on radiography, narrowing on radiography, erosion on radiography	1.058(.990–1.13) 5.31(2.196–12.85) 5.359(2.520–11.40) 4.435(2.022–9.728) 3.516 (1.682–7.348) 2.185(1.064–4.486) 2.582(1.157–5.765) 1.037(1.059–4.08) 2.192(1.037–4.63) 5.167(2.286–11.6) 3.778(0. 901–15.8) 3.821(1.713–8.523) 3.250(1.187–8.90) 2.215(1.005–4.882) 2.276(0.98–5.27)	0.01 0.001 0.001 0.001 0.001 0.03 0.02 0.05 0.04 0.001 0.05 0.001 0.02 0.05 0.05	calcinosis on PE muscle tenderness flexion contracture on PE	1.11 1.09 1.08	3.04(1.28–7.21) 2.99(1.21–7.40) 2.95(1.11–7.87)	0.01 0.02 0.03

* OR (95%CI): odds ratio, CI: confidence interval.

** Duration from non-Raynaud’s symptoms to time of taken radiography

# PE: physical examination

** FC: flexion contracture,

¶¶ TFRs: tendon friction rubs.

ŧ DLCO: diffusing capacity of the lung for carbon monoxide .

ŧŧ TOPO I abs: Topoisomerase I antibodies

### Serological and auto-antibodies findings

**Table 2** presents serological and auto-antibodies findings. In the current study, arthritis showed no association with anti-CCP and ant-RF (p=0.85 and p=.0.60, respectively).

### Radiographic pattern in two subtypes

Joint erosion was detected in 31 (17.2%) patients and joint space narrowing in 38 (21.5%) patients. Arthritis in radiography defined as the presence of both joint erosion and narrowing was seen in 18 (10.0%) patients. Eighty-seven (48.3%) patients had acro-osteolysis on radiography and resorption of bone was observed in 11 (6.1%). Calcinosis and flexion contracture on radiography was detected in 38 (21.1%) and 34 (18.8%) patients, respectively. Subgroup analysis of radiographic findings showed acro-osteolysis and flexion contractures were significantly more prevalent in patients with dcSSC (p=0.007 and p=0.05, respectively) (**Table 4**).

**Table 4.** Radiological findings in 180 SSc patients with different disease subsets.

	**All No(%)**	**Diffuse No (%)**	**Limited No (%)**	**P value**
**Joint pattern**				
**Erosion**	**31(17.2)**	16(51.6)	15(48.4)	0.88
Wrist	14/31 (45.1)	5(35.7)	9(64.3)	0.18
^¶^MCP	6/31(19.3)	3(50)	3(50)	0.89
^*^PIP	12/31(38.7)	8(66.7)	4(33.3)	0.32
^ŧ^DIP	8/31(25.8)	3(37.5)	5(62.5)	0.37
**Joint space narrowing**	**38(21.5**)	20(52.6)	18(47.4)	0.98
Wrist	11/38(31.4)	6(54.5)	5(45.5)	0.90
MCP	10/38(26.3)	6(60)	4(40)	0.63
PIP	24/38(63.1)	13(54.2)	11(45.8)	0.88
DIP	18/38(47.4)	10(55.6)	8(44.4)	0.83
**Arthritis**	**18 (10.0)**	11(61.1)	7(38.9)	0.45
**Bone pattern**				
Bone resorption	87(48.3)	55(63.2)	32(36.8)	0.007
Acro-osteolysis	87(48.3)	55(63.2)	32(36.8)	0.007
Resorption of distal ulna	11(6.1)	6(54.5)	5(45.5)	0.90
**Soft tissue pattern**				
Calcinosis	38(21.1)	22(57.9)	16(42.1)	0.47
Flexion contracture	34(18.8)	23(67.6)	11(32.4)	0.05

¶ MCP: metacarpophalangeal joints.

* PIP: proximal interphalangeal joints. DIP:

ŧ distal interphalangeal joints

### Association of radiographic findings with clinical and paraclinical features of SSc

Findings of hand radiography in univariate and multivariate analyses are summarised in **Table 5**. The results indicate that disease duration was correlated with joint erosion, bone pattern (acro-osteolysis and resorption distal ulna), and soft tissue pattern (calcinosis and radiological flexion contracture). Interestingly, arthritis was not associated with clinical or serological features of the disease. Acro-osteolysis was correlated with the diffuse subtype of the disease, the presence of TFRs, positive serology for anti-TOPO abs and disease duration.

**Table 5.** Association of radiographic findings with clinical and para-clinical features of SSc.

	**Univariate analysis**	**OR (95%CI)**		**Multivariate analysis**	**B**	**Odds (95%CI)**	**p**
Joint erosion on radiology	Raynaud’s’ phenomenon Telangiectasia Calcinosis on PE Tendon friction rub Time interval from Non-Raynaud’s to radiology	1.23(1.14–132) 3.11(1.03–9.39) 2.35(1.05–5.27) 2.27(0.98–5.27) [1.19(1t .00–1.25)	0.05 0.04 0.04 0.05 0.05	Disease duration	1.10	[1.10(1.03–1.18)	0.006
Joint space narrowing	Digital Ulcer/gangrene Calcinosis Flexion contracture on PE Advanced lung fibrosis HCT<30% Time interval from non-Raynaud’s to radiology	1.85(0.88–3.89) 4.52(2.11–9.66) [3.78(1.62–8.87) 2.55(1.22–5.32) [2.66(1.44–4.90) [1.07(1.01–1.15)	0.01 0.001 0.001 0.01 0.006 0.04	Calcinosis advanced lung fibrosis HCT<30%	1.53 0.81 1.25	4.65(2.10–10.3), 2.23(0.99–5.03) 3.50(1.03–11.86)	0.001 0.05 0.05
Joint arthritis on radiography	Calcinosis on exam Tendon friction rub Muscle pain Muscle atrophy HCT<30% Elevated aldolase	3.15(1.17–8.50) 3.25(1.18–889) 3.67 (1.33–10.1) 2.80(1.04–7.51) 3.36(1.28–8.86) 7.58(1.14–50.1)	0.02 0.02 0.008 0.04 0.02 0.02	no variable showed dependent association with arthritis on radiography	-	-	-
bone demineralization	Subtype of disease Digital ulcer-gangrene Calcinosis in PE Flexion contracture on PE Radiological flexion contracture Radiological acro-osteolysis Radiological patter-bone resorption anti-topo	2.680(1.459–4.923), 2.151(1.101–4.202) 2.534(1.231–5.218) 2.446(1.319–4.536) 3.553(1.455–8.672) 4.51(6.830–30.820) [1.084(1.025–1.147) 2.199(1.121–4.314)	0.001 0.02 0.01 0.004 0.004 <0.0001 0.012 0.011	acro-osteolysis	2.388	10.90(4.75–24.98)	0.001
Acro-osteolysis	Diffuse subtype of disease Telangiectasia Digital ulcer/gangrene Calcinosis in PE Tendon friction rub flexion contracture on PE Muscle atrophy Muscle pain Esophageal involvement Advanced lung fibrosis Anti-TOPO I Disease duration mRSS	2.27(1.25–4.14), 2.37(1.20–4.67) 2.30(1.20–4.41), 4.76(2.27–10.0), 5.16(2.28–11.6), 3.92(2.07–7.40), 2.25(1.19–4.24), 3.17(1.45–6.92) 2.03(1.02–4.05) 2.14(1.13–4.05), 3.92(2.07–7.40), 1.17(1.09–1.26) 1.05(1.01–1.09)	0.007 0.01 0.01 0.001 0.001 0.001 0.01 0.003 0.04 0.02 0.03 0.001 0.01	diffuse subtype of disease tendon friction rub anti-TOPO I disease duration	0.91 1.42 1.06 2.10	2.48(1.06–5.85) 4.14(1.60–10.7 2.90(1.19–7.10) 1.23(1.12–1.36)	0.04 0.004 0.02 0.001
Resorption of distal ulna	Oesophageal involvement Advanced lung fibrosis Disease duration from non- Raynaud’s to time of study	1.09(1.03–1.15) 3.93(1.10–14.0) 1.12(1.02–1.23)	0.04 0.02 0.02	Disease duration	0.11	1.11(1.01–1.22)	0.03
Calcinosis	Telangiectasia Calcinosis on PE Disease duration	2.45(0.99–6.34), 5.26(2.45–11.3), 1.63(1.08–1.25),	0.05 0.001 0.001	disease duration	0.13	1.143(1.06–1.23)	0.001
Flexion contracture	Diffuse subtype of disease Telangiectasia Calcinosis on PE Flexion contracture on PE Tendon friction rub Advanced lung fibrosis PAP> 40 HCT<30% ESR>30 TOPO I Muscle weakness Time interval of non-Raynaud’s phenomenon mRSS	2.14(0.97–4.72) 3.56(1.18–10.6) 7.12(3.17–15.9) [6.31(2.31–17.2) [3.82(1.71–8.52), [2.12(0.99–4.56) 2.67(1.07–6.68) 4.42(2.58–7.56) 2.67(1.21–5.86) 3.09(1.01–8.65) 2.41(1.11–5.23) 1.10(1.03–1.19) 1.06(1.02–1.11)	0.05 0.02 0.001 0.001 0.001 0.05 0.03 0.001 0.01 0.03 0.02 0.004 0.006	Disease duration mRSS calcinosis on PE HCT<30%	0.12 0.06 1.40 2.71	1.29(1.03–1.24) 1.06(1.01–1.12) 4.08(1.45–11.4) 15.1(3.70–60.9	0.01 0.01 0.01 0.001

* OR (95%CI): odds ratio, CI: confidence interval.

# PE: physical examination

** FC: flexion contracture,

¶¶ TFRs: tendon friction rubs.

ŧ DLCO: diffusing capacity of the lung for carbon monoxide (CO).

ŧŧ TOPO I abs: Topoisomerase I antibodies, mRss: modified Rodnan skin score

We also found DIP narrowing on radiography of 18 (10.0%) patients and DIP erosion in 8 (4.4%) patients. Narrowing coincided with erosion in 4 patients. Age group showed no significant relationship with DIP narrowing (p=0.62) or DIP erosion (p= 0.78), but there was a correlation between DIP narrowing and DIP erosion (Cramer’s V=0.28, p=0.04).

## DISCUSSION

As shown by results, the disease duration was a main dependent factor for developing joint erosion, acro-osteolysis, bone resorption, calcinosis and flexion contracture on hand radiography. Acro-osteolysis associated with more severe form of the disease (dcSSc subtype, the presence of TFRs, and anti-TOPO I), and was the only dependent variable associated with bone demineralisation.

In EUSTAR database, symptoms of arthritis were reported in 16% of patients and they were more prevalent in dcSSc than lcSSc (20% vs 13%), and arthritis detected on radiography in 18% of patients.^5,6^ Furthermore, muscle weakness and CPK elevation were reported in about 28.3% and 7.1% of all patients, respectively.^25^ Similar to the EUSTAR study, we detected muscle weakness in 28.1% of the patients.^6^ In our study, most patients with elevated CPK and aldolase were among those with muscle weakness, and muscle enzyme test can be useful as a diagnostic tool. In contrast to the previous study, in the current study, using both tests did not increase the diagnostic sensitivity of the test. ^26^

The prevalence of TFR in our study looks similar to Pittsburg’s study results (28%) as opposed to the results from the EUSTAR database (11%). This may be due to the presence of more patients with the diffuse subtype in the current and Pittsburg’s study compared to EUSTAR (52%, 49%, and 33%, respectively).^6,27^ In previous studies, the presence of tendon friction rubs was correlated with severe vascular, articular muscular and renal involvements,^7,18^ and TFRs were reported more often with FC on joints.^28^ Elevated ESR in patients with TFRs implicated the inflammatory nature of TFRs in scleroderma.^28^

In our study, we found RF in anti-CCP abs in 18.8% and 10.6% of the patients in the limited and diffuse subgroups. In a previous study, the prevalence of rheumatoid factor was reported in 27–30% of SSc patients and anti-cyclic citrullinated antibodies (anti-CCP abs) in 1.5 to 12%.^6,29,30^ Similar to other studies, RF and anti-CCP did not correlate with the clinical or radiological pattern of arthritis.^6,7^ Our results indicated a prevalence rate of (92.7%) anti-nuclear antibodies (ANA) and 10.4% ACA among patients. These numbers compared to the results from Liaskos et al., study with 97.5% ANA and 32.9% ACA positive patients were lower.^31^ In ANA negative patients, we should consider multiparametric autoantibodies testing, that could be helpful for early disease diagnosis. ^31^ Low level of ACA in our study was consistent with the results of two previous studies from Iran.^32,33^

We found anti-Topo isomerase I antibodies were more frequently associated antibodies with flexion contracture, bone demineralization, acro-osteolysis, in univariate analysis. In a large European multicentre study, the presence of anti-topo I antibodies show to be independently associated with tenosynovitis.^34^

In radiographic study, the prevalence of joint erosion has been reported between 5% and 40%.^15,20^ In our study, the prevalence of erosion, joint space narrowing, and arthritis were lower than those in the EUSTAR study report. In that study, 21% of SSc patients had articular erosion, 28.5% joint space narrowing, and 18% arthritis (erosion and joint space narrowing).^6^

In the current study, in a multivariate analysis, we found the time interval from non-Raynaud’s symptom to be a dependent variable associated with resorption of ulna, calcinosis, and flexion contracture on radiography. That could suggest the role that disease duration could play in progression of vasculopathy or the cumulative effect of trauma by time. Higher skin scoring and calcinosis were dependent factors for joint contracture. This can be explained by increased skin tightness and mechanical effect on developing erosion by time. In multivariate analysis, there was no association between arthritis on radiography and the factors that had meaningful correlations in univariate analysis. In a previous study, arthritis on radiological examination was associated with the cutaneous subset, CRP >10 mg/l, and disease duration.^6^

In our series, there was a low proportion of postmenopausal women (28%), and we found no significant difference in DIP erosion and narrowing according to age category. Besides, there was a correlation between DIP narrowing and DIP erosion, which suggests DIP erosion is not related to coincided OA. In a previous study, Avoca et al. suggest the possibility of DIP arthropathy as a SSc-related feature on radiology. However, they could not rule out findings with OA in the series, because most patients in the study were postmenopausal women.^6^ We found more increase in the prevalence of bone pattern compared to EUSTAR study (6% vs 2%, respectively).^6^

## LIMITATIONS

This study has some limitations. This was a point prevalence study of the clinical and radiographical findings; therefore, the results should be interpreted with caution, and causality cannot be interfered from the results. Muscle biopsy was not performed in patients with weakness and elevated muscle enzymes; thus, muscle weakness could not be interpreted as myositis. Likewise, elevated sPAP should not be considered as pulmonary hypertension.

## CONCLUSION

The study evaluated musculoskeletal involvement in SSc. Our findings indicate one-fifth of the patients have arthritis, muscle tenderness, and weakness on physical examination. CPK elevation that was accompanied with muscle weakness could be used as a diagnostic tool. Acro-osteolysis was seen with the severe form of the disease. We observed erosive arthritis on DIP, which may be considered as scleroderma-related arthropathy. Association of disease duration with radiologic findings could be an interdependent factor and suggestive of the cumulative effect of time on developing the lesion.
